# Engineering Inhalable Carboxymethyl Chitosan-Swellable Microgels for Pulmonary Delivery of Charged Hydrophilic Molecules †

**DOI:** 10.3390/gels11121015

**Published:** 2025-12-17

**Authors:** David Encinas-Basurto, Kiley McCombs, Ernest L. Vallorz, Maria F. Acosta, Rick G. Schnellmann, Heidi M. Mansour

**Affiliations:** 1 Biopolymers, Research Center for Food and Development, A.C., Hermosillo 83304, Sonora, Mexico; 2 Nanotechnology Program, Department of Physics, Universidad de Sonora, Hermosillo 83000, Sonora, Mexico; 3 Skaggs Pharmaceutical Sciences Center, The University of Arizona R. Ken Coit College of Pharmacy, 1703 E Mabel St., Tucson, AZ 85721, USA; 4 College of Medicine, The University of Arizona, Tucson, AZ 85721, USA; 5 Center for Translational Science, Florida International University, Port St. Lucie, FL 34987, USA; 6 College of Public Health & Social Work, Florida International University, Miami, FL 33199, USA; 7 Herbert Wertheim College of Medicine, Florida International University, Miami, FL 33199, USA; 8 College of Engineering & Computing, Florida International University, Miami, FL 33174, USA

**Keywords:** biodegradable biocompatible polymers, dry powder inhalers, therapeutic aerosols, human cells, suramin, leucine, in vitro, lung, respiratory

## Abstract

Swellable microparticles are a promising strategy for pulmonary drug delivery. They provide good aerosol performance in the dry state and enlarge after deposition in the lungs. In this study, we aimed to develop and characterize spray-dried microparticles composed of carboxymethyl chitosan (CMC), L-leucine, and suramin, a hydrophilic polyanionic drug. Microparticles were obtained by co-spray drying (Co-SD) formulations with increasing leucine content (0–10% *w*/*w*) and evaluated for morphology, thermal behavior, crystallinity, swelling, aerodynamic deposition using a Next Generation Impactor (NGI), and cytocompatibility in pulmonary epithelial cells. The 10% leucine formulation produced the highest fine particle fraction (35.2 ± 1.1%) and the lowest mass median aerodynamic diameter (1.0 ± 0.4 µm). These values indicate efficient in vitro deep lung deposition. XRPD and DSC showed that the Co-SD formulations were predominantly amorphous. Hydration studies revealed rapid water uptake and a clear increase in particle size, leading to the formation of swollen microgels. Cell viability assays demonstrated >85% viability up to 100 µM suramin, suggesting that CMC–leucine microgels enable efficient pulmonary delivery of hydrophilic drugs by combining respirable dry-state properties with in situ swelling and reducing immunological clearance. Future in vivo studies will be needed to assess long-term stability, macrophage interaction, and the translational potential of this delivery system.

## 1. Introduction

Pulmonary drug delivery is a well-established route for both local and systemic therapeutic administration, with benefits including a rapid onset of action, high bioavailability, and the avoidance of hepatic first-pass metabolism. The lungs’ enormous surface area, thin epithelial barrier, and considerable vascularization make them a desirable target for the treatment of chronic and acute respiratory disorders. However, efficient transport to the deep lung remains a challenge due to physiological limitations such as mucociliary clearance, enzymatic breakdown, and fast phagocytosis by alveolar macrophages [1,2].

Due to their portability, stability, and lack of propellants, dry powder inhalers (DPIs) are frequently employed for pulmonary administration [3,4]. Particles with aerodynamic sizes ranging from 1 to 5 µm are ideal for deposition in the alveolar area [5]. Alveolar macrophages, however, can easily clear particles in this size range, which reduces how long they remain in the lungs and limits their therapeutic effectiveness. One interesting approach to overcome the limitation is to use swellable microparticles. Particles in the respirable size range are rapidly eliminated by alveolar macrophages, which reduces lung retention. Because swellable microparticles deposit effectively when dry but expand after contact with lung fluids, changing to sizes less susceptible to phagocytosis, they have been offered as a solution to this challenge [6].

The majority of described systems have mostly concentrated on lipophilic or neutral compounds and relied on either PLGA, gelatin, or unmodified chitosan matrices, despite significant advancements in swellable microparticle design [6,7]. Furthermore, although L-leucine is known to enhance aerosol performance through surface crystallization, it has not been used in conjunction with an ionizable, hydrophilic polymer like carboxymethyl chitosan (CMC) to accomplish both aerodynamic control and post-depositional swelling within the same particle. Therefore, the current study presents a dual-function microgel system in which leucine enhances dispersibility and dry-state stability, while CMC facilitates hydration-driven swelling and prolonged release. By filling the gap between leucine-modified dispersible powders and swellable hydrogel carriers, this development expands the concept to polyanionic drugs like suramin, which typically have poor lung retention because of rapid breakdown and dissolution.

When these systems come into contact with lung fluid, they swell to produce larger hydrogel-like structures that can escape macrophage intake and prolong drug residency in the lung. In the dry state, these systems are designed to remain within the respirable aerodynamic size range [8,9]. Lee, Lee, Kim, Jung, Choi, Jeong, Jeong, Lee and Youn [6] produced inhalable microgels containing nintedanib and pirfenidone, demonstrating that post-depositional swelling enabled the particles to avoid alveolar macrophage phagocytosis while preserving high biocompatibility. CMC is a biocompatible, biodegradable, and mucoadhesive polysaccharide derived from the partial substitution of chitosan’s amino groups with carboxymethyl groups, improving its solubility under physiological pH. Due to its hydrophilic and polyelectrolyte nature, CMC exhibits a strong capacity to absorb water and form swollen hydrogel networks, making it particularly attractive for the design of sustained-release drug delivery systems. Upon hydration, CMC chains expand and reorganize into a hydrated matrix that can modulate drug diffusion [10].

Furthermore, the current platform differs from previously described spray-dried systems based on CMC or leucine in three additional ways. First, compared to the neutral or weakly basic drugs usually investigated, suramin poses a significantly higher encapsulation and retention difficulty due to its high hydrophilicity and strong polyanionic nature. Second, this formulation establishes a functional connection between dispersibility, swelling, and the potential for decreased macrophage clearance by providing a mechanistic coupling between dry-state aerodynamic control and hydration-triggered dimensional change. Lastly, a kinetic study of both Korsmeyer–Peppas and Peppas–Sahlin models is integrated in this work, providing mechanistic understanding of diffusion and polymer relaxation contributions for CMC-leucine microgels that have not been reported before.

Suramin, a hydrophilic and highly charged polyanionic molecule with therapeutic potential in inflammatory disorders, cancer, and parasitic diseases, was encapsulated in this study using CMC as the basic polymer by Co-SD, since suramin’s fast elimination and poor membrane permeability limit its clinical application despite its promising efficacy [11]. We aimed to increase suramin pulmonary bioavailability, facilitate local controlled release, and extend its residence time in the alveoli by encapsulating it in a swellable CMC matrix. L-leucine was also added to the formulation to enhance its dispersibility and aerodynamic qualities, ensuring effective delivery to the deep lung.

## 2. Results and Discussion

### 2.1. Morphology and Swelling Behavior of Spray-Dried Microparticles

**Figure 1:**
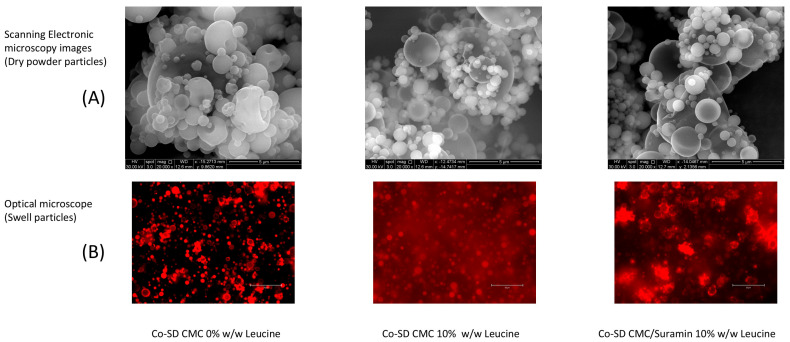
Dry-state morphology and swelling behavior of spray-dried CMC microgels. (**A**) SEM images; scale bar: 5 µm. (**B**) Optical microscopy after hydration shows the transition of spray-dried particles into hydrated microgels; scale bar: 50 µm. Reprinted from Encinas-Basurto et al. [13].

The simultaneous study of particle morphology in the dry state and their behavior after hydration provides significant insights into the functionality of Co-SD microgels designed for pulmonary administration. SEM analysis of our formulations indicated a noticeable change in structural organization as leucine concentration increased (Figure 1). Microgels formed without leucine (Co-SD CMC 0% Leucine) had unbalanced, collapsed morphologies with smooth surfaces and significant aggregation. These characteristics are typical of systems lacking surface-active stabilizers, and they are frequently associated with low dispersibility, high cohesion, and limited aerodynamic performance [12]. In contrast, formulations containing 10% leucine, both with and without suramin, had particles that were more spherical, with prominent surface corrugation and porous features.

The optical microscopy images (Figure 1B) clearly show that the particles rapidly changed into swollen microgels upon hydration. In comparison to their dry form, the hydrated structures exhibited a significant increase in diameter, with an estimated expansion from 0.5 ± 0.3 µm to 5.5 ± 2.4 µm, corresponding to a volumetric increase of approximately tenfold. This dimensional change is in accordance with the swelling behavior observed for comparable hydrogel-based microgels, Du, El-Sherbiny and Smyth [7], and represents the relaxation and water uptake capability of the CMC-GNP network. In addition to being structurally expected for CMC-based systems, this post-depositional swelling is functionally significant since it could increase the hydrated microgels above the alveolar macrophage uptake threshold while maintaining a hydrated environment for prolonged suramin release [6].

The surface crystallization of leucine during the drying process is responsible for the characteristic corrugated shape of the spray-dried particles. The creation of a semi-rigid shell as a result of this phenomenon, which has been extensively reported in the literature, reduces particle collapse and lowers bulk density, two characteristics that are ideal for enhancing aerosolization in pulmonary delivery systems [14]. These findings are consistent with the work of Wang et al. [15], who found that leucine-induced wrinkling improved dispersibility in swellable pulmonary microparticles. Huang et al. [16] found that leucine can reduce powder cohesion from 4 kPa to 1 kPa while also improving surface morphology, allowing for optimal deep lung delivery. These effects become apparent when surface coverage surpasses 60–70%, improving dispersibility similar to our 10% *w*/*w* leucine formulations.

In addition to these findings, the swelling behavior observed under an optical microscope emphasizes the microgels’ dual functioning. When exposed to aqueous conditions, leucine-containing particles rapidly absorbed water and visibly increased in size. Their shapes became translucent and cloudy, which is a typical indicator of hydrogel production. This alteration was most noticeable in the 10% leucine formulations, which transformed into soft, hydrated structures in minutes, in contrast to the non-leucine formulation, which retained its compact, collapsed appearance with small size change. The fact that swelling was not impeded despite the presence of a hydrophobic, crystalline leucine shell supports previous findings that wrinkled morphologies facilitate rather than restrict water infiltration. The incorporation of GNP as a natural crosslinker was crucial to the CMC matrix’s stability due to its interaction with the primary amino groups of CMC through a mild nucleophilic substitution, creating strong amide bonds that enhance the microgel network and preserve biocompatibility [17,18]. Previous studies have shown that GNP-mediated stabilization improves the durability and safety of chitosan-based systems [19].

For instance, Chen et al. [20] demonstrated that the surface crystalline layer formed by leucine during spray drying did not hinder water penetration, and particles maintained hydration responsiveness and functional porosity upon aerosolization

This is confirmed by the findings of Wang et al. [21], who reported comparable behavior in inhalable spray-dried particles with roughened surfaces, stressing that increased surface area and porosity facilitated capillary-driven hydration. Laser diffraction experiments indicated swelling in the CMC/Sur 10% Leucine formulation, with hydrated particle sizes exceeding the 8 µm threshold and Geiser [22] and Lee, Lee, Kim, Jung, Choi, Jeong, Jeong, Lee and Youn [6] found that particles larger than 5–10 µm are less likely to be recognized and phagocytized by alveolar macrophages. Laser diffraction was used to measure particle size distributions to confirm the observed swelling tendencies quantitatively. Volume-based diameter measurements revealed that the median particle size (X_50_) started from 3.2 µm in the Co-SD CMC 10% leucine formulation to 8.56 µm in the Co-SD CMC/Sur 10% Leucine system, indicating significant swelling behavior. The X_90_ value increased from 8.83 µm to 25.58 µm for the formulations, indicating the production of large, hydrated structures in the pulmonary environment. Notably, the span values, which reflect the width of the size distribution, increased (from 1.87 to 2.41), indicating more swelling heterogeneity due to varied drug–polymer interactions and particle porosity.

Although CMC and leucine have individually been studied in spray-dried inhalable systems, their combination within a single swellable microgel platform for pulmonary delivery of highly charged hydrophilic drugs has not yet been reported. Previous studies using leucine mainly focused on its ability to migrate to the droplet surface during spray drying and to form a crystalline, corrugated shell that lowers particle cohesion and enhances fine particle fraction and stability [23,24]. Likewise, CMC-based inhalable powders have been investigated for their mucoadhesive and hydrophilic swelling properties; however, these studies did not incorporate a surface-modifying excipient to enhance aerosolization. Here, we combine the aerosol-enhancing properties of leucine with the swellability of CMC to design microparticles that are respirable in the dry state yet transform into hydrated microgels upon deposition, potentially minimizing alveolar macrophage uptake as particles enlarge beyond the 5–10 µm range [6].

Lee, Lee, Kim, Jung, Choi, Jeong, Jeong, Lee and Youn [6] developed 12 μm PEG–albumin microgels containing antifibrotic nanoparticles for pulmonary fibrosis, demonstrating similar macrophage phagocytosis-evading strategies. Their findings showed that alveolar macrophages poorly incorporate particles larger than the 6–10 μm threshold, which increases lung occupancy and lowers dosage frequency. The current CMC-leucine approach is supported by a similar physical mechanism, whereby post-depositional swelling produces hydrated structures beyond the region of macrophage engulfment, potentially improving retention and localized efficacy. Taken together, these findings suggest that using CMC as a swellable matrix and leucine as a surface-modifying excipient yields a system that transitions from highly dispersible, respirable particles in the dry state to volumetrically expanded, soft microgels after pulmonary deposition. This dual-phase activity not only improves aerodynamic delivery to the deep lung but also promotes a longer residence time by evading immune clearance and maintaining drug release.

### 2.2. Thermal and Structural Characterization of Spray-Dried Microparticles

**Figure 2:**
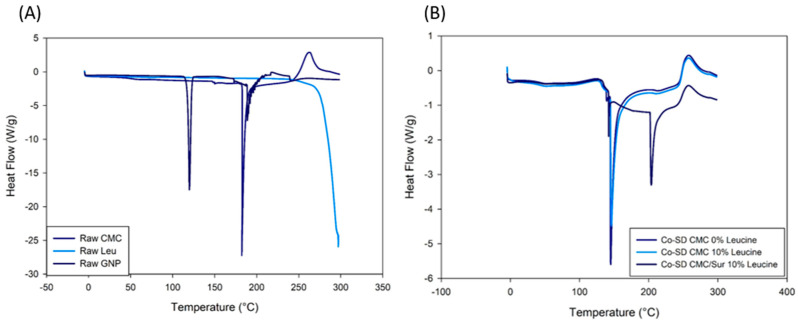
DSC thermograms of (**A**) raw materials and (**B**) spray-dried CMC-leucine formulations. Raw suramin and leucine exhibit characteristic crystalline melting peaks, whereas these transitions are absent in the co-spray-dried powders, indicating a predominantly amorphous solid state and molecular dispersion of the components within the CMC matrix.

The spray-dried microgels’ physical state and thermal transitions were examined using differential scanning calorimetry (DSC), X-ray diffraction (XRD), and hot-stage microscopy (HSM). These complementary approaches offered information about the crystallinity, molecular dispersion, and stability of the drug and excipients during spray drying. The raw materials’ crystalline nature was verified by DSC thermograms, which showed that leucine had a pronounced endothermic peak at 290 °C and suramin at 280 °C (Figure 2). These transitions were either eliminated or significantly reduced in the thermograms of the Co-SD formulations. The Co-SD CMC/Sur 10% Leucine formulations lack melting endotherms, suggesting that both the drug and leucine either become molecularly dispersed within the CMC matrix or change into an amorphous state [25]. In systems previously reported by Wang, Wan, Lu, Quan, Pan, and Liu [12], Co-SD leucine-modified chitosan particles resulted in the inhibition of crystalline thermal events, which were attributed to drug–excipient interactions and rapid solvent evaporation.

On the other hand, Figure 2B Co-SD formulations show notable variations in thermal performance. No melting peak for suramin appears in Co-SD CMC 0% Leucine, indicating that the drug was effectively amorphized or molecularly distributed throughout the polymer matrix during spray drying. The thermogram of Co-SD CMC/Sur 10% Leucine, which shows only broad transitions or slight endothermic shifts and no suramin or leucine melting peaks, gives additional support to this. These findings show amorphous solid dispersions and a loss of crystallinity, a change that is commonly observed in SD systems that use hydrophilic polymers such as CMC [26]. Suramin has many sulfonate (–SO_3_^−^) groups that can form hydrogen bond interactions with the polysaccharide backbone and electrostatic connections with the protonated amino sites (–NH_3_^+^) of CMC. GNP-crosslinked chitosan and CMC hydrogels loaded with polyanionic or hydrophilic pharmaceuticals have been shown to exhibit similar ionic and hydrogen bonding interactions [19,27].

Similar results have been observed by studies that have included leucine in Co-SD inhalation formulations, including those conducted by Mangal et al. [28] and Alhajj, O’Reilly and Cathcart [23]. Leucine surface crystallization during SD is frequently attributed to the absence of its melting peak. Furthermore, as indicated by the small variations in heat flow at 100–150 °C, drug–excipient interactions, such as hydrogen bonding between the amino or carboxymethyl groups of CMC and the sulfonate groups of suramin, can also inhibit or alter thermal transitions [19].

Amorphous pharmaceuticals are less thermodynamically stable than crystalline drugs and are more likely to recrystallize during storage, particularly in humid or high-temperature settings [29]. This topic has been addressed in the literature by promoting the use of surface crystallizing excipients, such as leucine, or selecting polymer matrices with high hydrogen bonding capability, both of which are utilized in the current formulation [30,31]. The lack of recrystallization evidence in the thermograms indicates that the CMC-leucine combination produces a stable amorphous matrix, at least under the heat settings studied.

**Figure 3:**
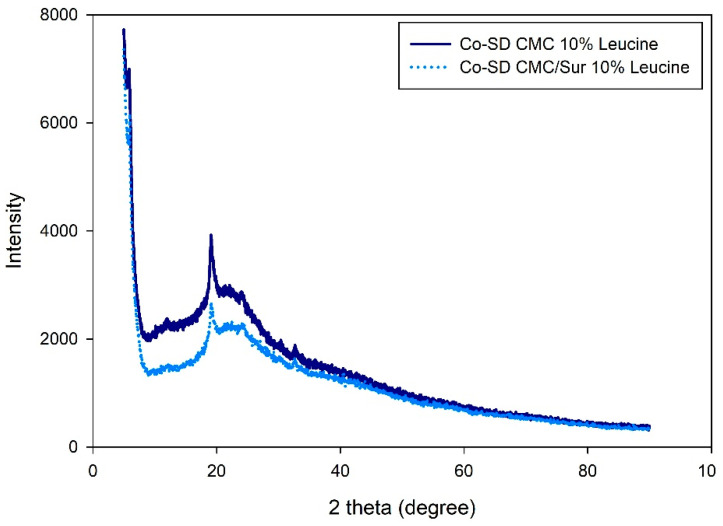
X-ray diffraction patterns of spray-dried CMC-leucine microgels with and without suramin.

Both Co-SD CMC 10% Leucine and Co-SD CMC/Sur 10% Leucine had broad halos devoid of sharp peaks that would indicate crystalline components, as seen in Figure 3. Amorphous polymers typically exhibit a halo centered about 20° at 2θ, which signifies a loss of long-range molecular order [32]. This is in line with research by Alhajj, O’Reilly and Cathcart [23], who also found that SD particles containing leucine exhibited better dispersion behavior and less crystallinity. The absence of crystalline reflections in the suramin-loaded formulation indicates that the drug was successfully molecularly dispersed throughout the polymeric matrix.

This change to an amorphous state is very important for pulmonary administration of drugs. Amorphous particles have higher apparent solubility and dissolution rates, which is useful in the deep lungs’ limited fluid environment. According to Paudel et al. [33] and Motzwickler-Németh et al. [34], SD increases the dissolution kinetics of weakly water-soluble drugs, resulting in increased bioavailability upon inhalation.

**Figure 4:**
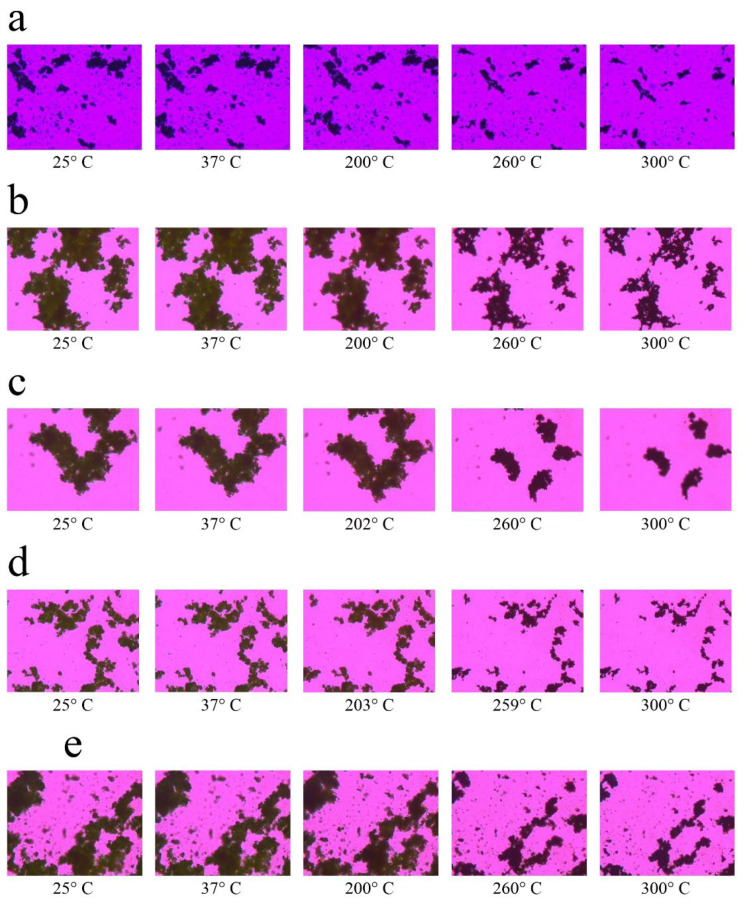
Representative hot-stage microscopy images showing thermal behavior of spray-dried CMC-based microparticles at increasing temperatures. Formulations include: (**a**) Raw CMC *w/v* 10% Leucine, (**b**) 5% Leucine, (**c**) 2.5% Leucine, (**d**) 0% Leucine, and (**e**) CMC/Sur *w/v* 10% Leucine. Progressive loss of birefringence and structural softening with temperature indicate the thermal transitions and amorphous nature of the co-spray-dried powders.

Finally, HSM analysis clearly confirmed the thermal transitions (Figure 4). Pure suramin and leucine melted and collapsed fast at their melting points, but SD microparticles relaxed and deformed gradually across a wider temperature range. This behavior, which occurs in the absence of distinct melting, supports the amorphous state and matches the behavior of gel-like matrices. Notably, particles containing leucine showed delayed morphological changes under heat, most likely due to the stabilizing action of the leucine surface layer, which delays structural relaxation and increases thermal resistance [35].

Since amorphous matrices often exhibit higher apparent solubility and faster dissolution than crystalline materials, the mainly amorphous properties observed in the Co-SD formulations by XRPD and DSC are relevant. This is effective in the alveolar region’s very low fluid conditions, where accessible liquid amounts are estimated to be only 10–20 μL [32]. Due to their increased free energy and decreased molecular packing, amorphous solid dispersions have been demonstrated in recent research to enhance dissolution performance in moisture-restricted environments [36,37]. However, during storage, amorphous phases can be vulnerable to recrystallization or relaxation induced by moisture and the interplay with the storage temperature relative to the glass transition temperature, T_g_ [38]. Although the addition of GNP crosslinking and L-leucine to our system could decrease hygroscopicity and limit molecular mobility, factors known to help in the stabilization of amorphous states require careful consideration.

Co-SD microparticle compositions behaved very differently under heat. Microparticles of Co-SD CMC 10% Leucine and Co-SD CMC/Sur 10% Leucine lacked a distinct melting point. Instead, they gradually melted and deformed at about 150–180 °C, without converting to a distinct liquid phase. Notably, the deformation process was slower in leucine-containing particles, indicating that leucine provides structural stiffness or thermal protection to the outer shell. According to Alhajj, O’Reilly and Cathcart [23], amorphous SD powders often have broad softening ranges rather than acute melting events in HSM, which corresponds to suppressed melting peaks in DSC. This behavior is favorable in the context of inhalation, where physical homogeneity and regulated phase transitions are desired to prevent particle melting or agglomeration during aerosolization with mild heating.

A higher leucine concentration in spray-dried CMC powders enhances morphological and thermal stability, as evidenced by delayed and progressive softening across a wide temperature range [23]. This feature is especially desirable for inhalation powders because it reduces the risk of particle fusion or agglomeration during aerosolization, resulting in consistent and effective drug administration [39]. In conclusion, the combined DSC, XRD, and HSM data show that the spray-dried CMC-based microparticles are amorphous and thermally stable under the studied conditions. This change is related to increased dissolving potential and favorable pulmonary deposition, although long-term stability during storage must be carefully monitored. These findings suggest that the formulation is an excellent option for inhaled drug delivery systems, striking a balance between increased performance and the need for solid-state stabilization.

### 2.3. Controlled Release of Suramin from Leucine-Modified CMC Microparticles

**Figure 5:**
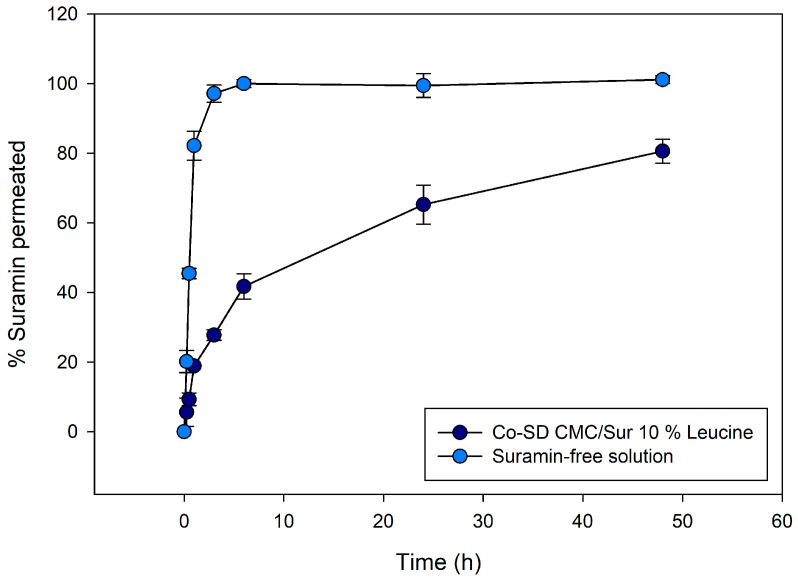
Cumulative release profiles of suramin over 48 h from co-spray-dried CMC/Leucine microgels (10% leucine) compared with free suramin solution. Microgels exhibit sustained release (ca. 80% at 48 h), whereas free suramin diffuses rapidly (100% within 6 h), highlighting the controlled-release capabilities of the swellable CMC matrix. Error bars represent mean ± SD (*n* = 3).

Figure 5 presents the cumulative permeation profiles of suramin across synthetic dialysis membranes over a 48 h period, comparing two formulations: suramin-free aqueous solution and suramin encapsulated in CMC microgels. The CMC-leucine microgels demonstrated a sustained release profile, releasing 41.7% at 6 h, 65.3% at 24 h, and 80.6% at 48 h. On the other hand, due to analytical variability, free suramin dissolved almost entirely during the first six hours, achieving 100% release at six hours and roughly 99–101% at later time points. The ability of the microgel matrix to considerably inhibit the release of a highly water-soluble polyanionic medication is demonstrated by this comparison.

The data reveal a clear contrast in drug release kinetics between the two systems. Approximately 90% of the drug permeated the free suramin solution within the first four to six hours, indicating an initial burst release. The CMC formulation, on the other hand, showed a noticeably slower and longer-lasting diffusion behavior. The amount of suramin that permeated the solution was significantly less than that of the free solution at early time periods (up to 6 h). However, drug release persisted steadily through the last time point (48 h), indicating that the polymeric matrix acted as a barrier that modulated the release. Instead of being weakly adsorbed or surface-bound, suramin was well-entrapped within the microparticles, as evidenced by the lack of a burst effect.

The absence of an initial burst release supports the idea of uniform drug trapping within the microgels, consequently avoiding surface localization. Suramin, which contains several sulfonic acid groups, has been shown to interact electrostatically with positively charged peptides and proteins [40]. Similarly, chitosan and its derivatives, including CMC, have demonstrated a high capacity for ionic complexation with anionic drugs and biomolecules, suggesting that this interaction may serve as a regulatory mechanism in drug release kinetics [41,42]. This superficial leucine layer may hinder initial water ingress, thereby delaying matrix swelling and the onset of drug release. This phenomenon has been reported in other leucine-based formulations, where surface crystallinity correlates with reduced wetting and delayed drug diffusion [20,43]. Upon hydration, however, this leucine-induced shell may facilitate deeper water penetration and matrix relaxation. The porous morphology, promoted by leucine during droplet drying, enhances internal diffusion pathways, enabling sustained release. Consequently, suramin embedded in internal domains can migrate outward more gradually, consistent with the observed dissolution profile.

Although our formulation employs leucine primarily as a dispersibility enhancer in microparticles for pulmonary delivery, the role of leucine in modulating drug release has also been studied. Alhajj et al. (2021) [23] demonstrated the effect of leucine in enhancing the dissolution of polyphenolics (curcumin, quercetin, and trans-resveratrol) from their ternary co-spray-dried particles by increasing the surface area of the corrugated particles, which made it feasible for more dissolving medium to come into contact with the particles. Additionally, the leucine matrix in which they were distributed enhanced the polyphenolics’ wettability and solubility, since leucine is soluble in water. However, in contrast to their study, leucine directly improved solubility; leucine appears to delay matrix hydration and drug diffusion in our system by inhibiting water input through partial surface crystallization.

To gain insight into the release mechanism, the experimental data were fitted to the Korsmeyer–Peppas (KP) empirical model, which describes the early stage of drug release from polymeric matrices according to the equation:

$$\frac{M_{t}}{M_{\infty }}=k\;t^{n}$$

where *M_t_/M∞* represents the fractional drug release at time *t*, *k* is the kinetic constant that includes structural properties, and *n* is the diffusional exponent that indicates the release mechanism. Fitting the current data to the KP model revealed *n* = 0.55 and *k* = 0.156 (R^2^ = 0.987), showing an anomalous transport mechanism dominated by diffusion, with modest contribution from polymer relaxation of the hydrated CMC-GNP matrix. These results are consistent with the morphological evidence of swelling in hydrated microgels.

An additional fitting using the Peppas–Sahlin model was carried out in order to better understand the mechanism, as explained below. The release profiles were further fitted to the Peppas–Sahlin (PS) model, which is expressed as follows, in order to further distinguish between the diffusion and relaxation components:

$$\frac{M_{t}}{M_{\infty }}=k_{1}\;t^{m}+k_{2}\;t^{2m}$$

where *m* is the diffusional exponent and *k*_1_ and *k*_2_ stand for the Fickian diffusion and relaxation contributions, respectively. Drug release is mostly diffusion-controlled, as confirmed by the fitted parameters, which produced *k*_1_ = 0.18, *k*_2_ = 0, and *m* = 0.40 (R_2_ = 0.987). Particle swelling was confirmed by optical microscopy, but the small *k*_2_ term indicates that swelling is not the rate-limiting step in these circumstances. Leucine-modified inhalable microgels and GNP-crosslinked chitosan have been shown to exhibit similar behavior, where morphological swelling takes place without appreciably changing the diffusion-dominated release mechanism [7]. Significantly, the presence of L-leucine in the spray-dried particles creates a hydrophobic and partially crystalline surface shell that momentarily prevents water from entering during the early hydration stage, contributing to the initial lag phase observed before the diffusion-driven release [15].

Whitehead and Kasapis [44] demonstrated similar results when simulating the transport of the hydrophilic molecule ascorbic acid through chitosan and gelatin matrices crosslinked with GNP. They discovered that Fickian diffusion can continue to be the dominant process even in swelling biopolymer networks, utilizing the Korsmeyer–Peppas and Peppas–Sahlin equations, particularly when the diffusing molecule is highly water-soluble and polymer relaxation is constrained by crosslinking. Their results corroborate our theory that the hydrophilic suramin molecule is primarily driven by diffusion through the hydrated polymer mesh rather than relaxation-controlled release, despite observable swelling in the CMC-GNP microgels.

Additionally, the findings from our study align with previous reports demonstrating the functional benefits of incorporating L-leucine into chitosan-based pulmonary formulations. In particular, Muhsin et al. [45] demonstrated that the amphiphilic particles that were produced by chemically conjugating L-leucine to chitosan improved dispersibility and facilitated extended drug release. Delayed hydration due to leucine content, prolonged suramin release, and surface morphological changes are comparable to those reported in conjugated systems, although our formulation is based on leucine physical integration.

### 2.4. Aerodynamic Evaluation with Next Generation Impactor (NGI)

The lung deposition efficiency of the formulations was determined using the NGI. Five formulations based on CMC with different proportions of L-leucine (0%, 2.5%, 5%, 10% *w*/*w*) and an additional formulation with incorporated suramin (CMC-Sur 10% *w*/*w* Leucine) were analyzed. The results showed a progressive improvement in the critical aerodynamic performance parameters as the proportion of leucine increased (Table 1). The mean aerodynamic diameter (MMAD) decreased significantly from 11.3 ± 1.8 µm in the formulation without leucine (CMC 0% Leucine) to 1.0 ± 0.4 µm in the formulation with 10% leucine (CMC 10% Leucine), an ideal value to reach the alveolar region of the lung (optimal range for lung deposition: 1–5 µm) [4,32].

The fine particle fraction (FPF) also increased significantly, rising from 13.5 ± 1.8% in CMC 0% Leucine to 29.4 ± 0.9% in CMC 5% Leucine while staying high at 27.0 ± 3.4% in CMC 10% Leucine. The formulation with 10% leucine had a maximum value of 85.3 ± 1.3% for respiratory yield, an accurate measure of the percentage of dosage that may reach deep lung regions. A high FPF value is preferred in DPIs because it correlates with the effectiveness of pulmonary drug delivery, particularly to the deep lungs. The European Medicines Agency (EMA) and the Food and Drug Administration (FDA) typically consider FPFs above 20% indicative of satisfactory commercial formulation deposition performance [46].

Our findings enhance these values and are equivalent to or superior to those of other inhalable drug delivery systems described in the literature. For example, Ni, Zhao, Liu, Liang, Muenster and Mao [9] created chitosan-based swellable microparticles embedded with nanocrystals and reported FPFs of 25–30%, whereas Yan et al. [47] achieved FPFs of approximately 39% in hydrogel-based microparticles. However, their MMAD was larger (5.68 µm), suggesting deposition in bronchi instead of alveoli. Previous studies have shown that L-leucine acts as a surface modifier during spray drying, encouraging the formation of particles with low interparticle cohesiveness, rough morphology, and enhanced dispersibility [15,23].

**Figure 6:**
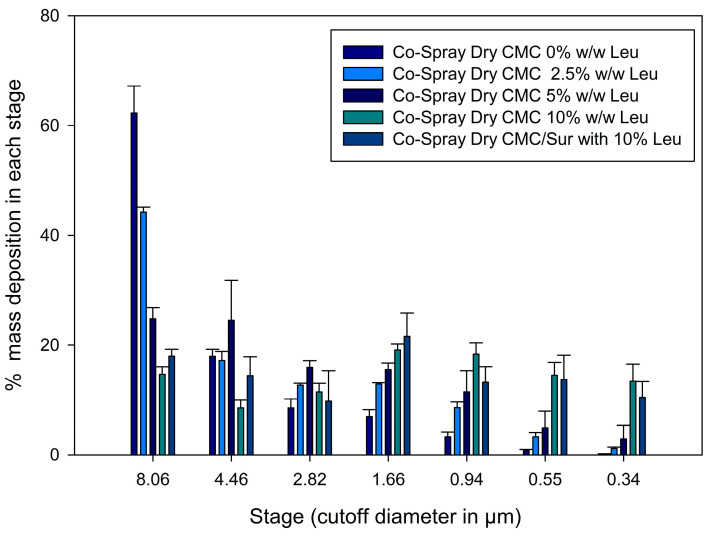
Aerodynamic deposition profiles of spray-dried CMC microparticles with varying leucine concentrations using a Next Generation Impactor (NGI); error bars represent mean ± SD (*n* = 3). Reprinted from Encinas-Basurto, McCombs, Vallorz and Mansour [13].

Figure 6 displays the cumulative % deposition profile at each NGI stage. A significant amount of large (>8 µm), non-respirable particles were found in the early stages (stages 1 and 2) of the formulation without leucine (CMC 0% Leucine). In contrast, formulations with leucine demonstrated a gradual transition to deposition in deeper levels (stages 3–6), linked with particles with MMAD within the optimum range (1–5 µm).

In particular, the 10% leucine formulation showed an optimal aerodynamic distribution to reach the pulmonary alveoli by concentrating its deposition between stages 4 and 6 (about 2.1 to 1.1 µm). The previously mentioned increase in RF and FPF is directly correlated with this redistribution of deposited mass, with the highest deposition in the 1.36 to 0.98 µm stages—the ideal size to get past mucociliary clearance and deposit in the alveoli. Furthermore, this kind of smart particle may avoid alveolar fast clearance. According to Geiser [22], particles larger than 0.5 µm, yet having a swellable shape or low density, can evade effective phagocytosis. Accordingly, new approaches, such as the use of effervescent microparticles, have demonstrated that altering particle behavior in moist lung environments enhances the efficacy and retention of therapy [21].

Recent findings indicate that alveolar macrophage clearance is highly dependent on the physical characteristics of inhaled particles, including stiffness, porosity, and post-depositional swelling. According to Liu, Guan, Qin, Zhang and Mao [38], particles with low stiffness or swellability behavior can evade phagocytosis more successfully than rigid, spherical particles. Significantly, recent research on inhalable hydrogel-based microgels by Lee, Lee, Kim, Jung, Choi, Jeong, Jeong, Lee and Youn [6] has demonstrated that particles that are originally aerosolized-sized (1–2 µm) can swell in the pulmonary environment to sizes > 10 µm, surpassing the alveolar macrophages’ phagocytic capacity. Even after 72 h, these microgels demonstrated limited macrophage uptake and sustained lung retention in their study. This post-depositional swelling mechanism is essential because, although the particles must be small enough to enter the deep lung during inhalation, their subsequent expansion prevents macrophages from internalizing them, extending the duration of drug residency and enhancing the local therapeutic effect of microgel-based inhalable formulations.

Yan, Wu, Miao, Ren, Wu and Shen [47] found that hydrogel microparticles with aerodynamic sizes ranging from 3.45 to 6.48 µm were mostly deposited in the bronchial region, restricting their reach to the alveolar space and lowering the potential for deep lung therapeutic activity. In contrast, Lee, Lee, Kim, Jung, Choi, Jeong, Jeong, Lee and Youn [6] studied humidity-responsive microgels with initial sizes of ~1–2 µm that swell when exposed to pulmonary environment. These microgels accumulated preferentially in the alveoli, allowing for prolonged lung retention of up to 72 h with negligible absorption by alveolar macrophages. Formulations like ours in this study, which include L-leucine to enhance aerodynamic diameter and utilize swellable microgel structures, are ideally positioned to produce favorable biodistribution profiles, longer residence times, and improved therapeutic efficacy in the deep lung.

The translational significance of the novel formulation is further demonstrated through contrasts with currently available DPI products. FPF values in the range of 20–30% are consistently delivered by the majority of commercial DPIs, including popular devices like Breezhaler^®^, Turbuhaler^®^, and HandiHaler^®^. FPF values above 20% are generally considered the standard for adequate lung deposition and therapeutic efficacy. In addition to achieving these established commercial benchmarks, the 10% leucine formulation’s FPF of 35.2% indicates a notable improvement in aerosolization efficiency. The formulation’s strong potential as a competitive inhalable system for pulmonary drug delivery is supported by this performance, which surpasses what is usually seen for marketed DPIs [48,49]. This level of aerosolization efficiency indicates that the CMC–leucine microgels perform within, and in some cases exceed, the FPF range associated with viable commercial inhalation systems, suggesting they have the potential to be a competitive inhalable platform for pulmonary drug delivery.

Achieving deep lung targeting and avoiding rapid immune elimination are two significant challenges in pulmonary drug administration that are addressed by this dual-function design, aerodynamic during inhalation and anti-clearance post-deposition. Additionally, these intelligent microparticle systems are highly adaptable. They can be modified to deliver various types of therapeutic agents, including proteins, peptides, and small, highly water-soluble molecules, such as suramin. By combining structural design (microgels) with surface engineering (L-leucine coating), this strategy holds strong potential for developing next-generation inhalable therapies that are both effective and patient-friendly.

### 2.5. Cytotoxicity Studies

**Figure 7:**
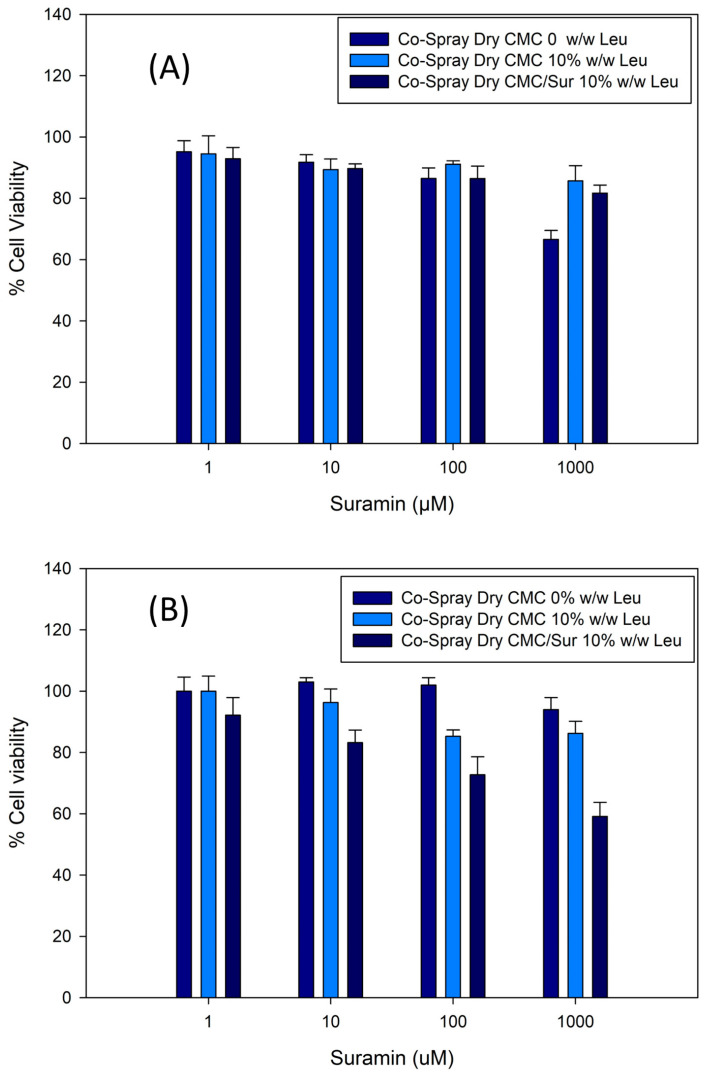
Cell viability of pulmonary epithelial cells exposed to spray-dried CMC microparticles containing various concentrations of suramin (1–1000 µM). (**A**) H441 and (**B**) H358. Formulations maintained high viability (>80%) across the tested range, indicating good cytocompatibility. Error bars represent mean ± SD (*n* = 3).

The cytotoxicity of the Co-SD formulations was assessed by determining the viability of pulmonary epithelial cells exposed to increasing doses of suramin, both free and encapsulated in CMC-based microgels containing L-leucine. Figure 7 demonstrates that all formulations are highly biocompatible at low and intermediate concentrations (1–100 µM), with cell survival exceeding 85%. The maximum concentration tested (1000 µM) resulted in a dose-dependent decrease in viability, particularly in the Co-Spray Dry CMC/Sur 10% Leucine formulation, where viability reduced to around 60–65%.

Notably, formulations without suramin maintained viability above 90% across all tested doses, demonstrating that the CMC polymer matrix and L-leucine excipient are not innately toxic. These findings suggest that the observed toxic effects at higher dosages are primarily due to suramin, not the delivery mechanism.

Importantly, encapsulating suramin in microgels appears to reduce its cytotoxicity at moderate dosages. This behavior is likely caused by controlled release from the microgel matrix, which decreases the rapid exposure of cells to high concentrations of the drug, as described for other inhalable polymer-based systems.

Lee, Lee, Kim, Jung, Choi, Jeong, Jeong, Lee and Youn [6] reported similar protective effects in inhalable microgels composed of biocompatible polymers that contained the hydrophilic drugs nintedanib and pirfenidone. In their study, cell viability remained above 85% at therapeutic concentrations, and the microgel system proved to mitigate the acute toxicity of free drugs. The similarity between these experiments highlights the benefits of microgel-based pulmonary delivery methods in reducing local cytotoxicity, particularly for hydrophilic or charged drugs, such as suramin. Furthermore, the use of CMC as a mucoadhesive and swellable matrix may enhance cytocompatibility by forming a hydrated barrier that regulates drug distribution and lowers cellular stress. Overall, these findings show that CMC-leucine microparticles are well tolerated by lung epithelial cells, indicating their potential as a biocompatible and safe delivery route for local pulmonary administration of hydrophilic drugs.

## 3. Conclusions

This study effectively created Co-SD microparticles based on CMC, L-leucine, and suramin. Leucine improved aerodynamic efficiency by decreasing particle cohesion and increasing dispersibility. Karl Fischer titration showed that the residual water content increased as the leucine concentration increased. It increased from 16.75 ± 0.50% in formulations without leucine to 23.63 ± 1.37% at 10% leucine. Interestingly, the water content decreased to 17.77 ± 0.85% when the model hydrophilic drug suramin was added to the 10% leucine formulation. This suggests that the drug and matrix may interact to influence water retention. The formulations with 10% *w*/*w* leucine had the lowest MMAD and the highest fine particle fraction (FPF), indicating that they were suitable for inhalation therapy. Hydration enables rapid particle expansion, resulting in soft, hydrated microgels, as demonstrated by optical imaging and laser diffraction measurements. This implies that the CMC-leucine microgels may increase therapeutic efficacy by extending their residence duration in the lung by delaying their rapid removal. Collectively, these findings demonstrate that CMC-leucine microgels combine respirable dry-state properties with rapid post-depositional swelling, enabling the potential to evade macrophage clearance and prolong lung residence. Future studies involving in vitro macrophage uptake and in vivo deposition experiments will be conducted to confirm these effects and evaluate therapeutic outcomes in chronic pulmonary disease models. Overall, these findings suggest that the CMC-leucine microgel system has potential for effective lung deposition and sustained release, with a probable decrease in macrophage clearance resulting from particle swelling. However, further research on macrophage interaction and in vivo retention should confirm this assumption.

## 4. Materials and Methods

### 4.1. Preparation of Advanced Spray Drying and Co-Spray Drying in Open Mode Using Organic Solvent

CMC (MW: (249.12)*n* DD = 90%, Santa Cruz Biotechnology, Inc., Santa Cruz, CA, USA), Sur (10% *w/w* CMC), and Genipin (GNP) (1:0.1, CMC/GNP) (Sigma Aldrich, St. Louis, MO, USA) were dissolved in a 30% EtOH solution at 50 °C. Prior atomization using a Büchi advanced mini spray dryer B-290, coupled with a Büchi inert loop B-295 and a high-performance cyclone (Büchi Labortechik AG, Flawil, Switzerland), was performed. Different amounts of L-Leucine (2.5, 5, and 10% *w*/*w* of CMC) were added to the nano-/microgel suspension. Spray drying was performed in closed mode using UHP nitrogen gas as the atomizing gas and a 0.7 mm diameter stainless steel nozzle. A CMC/GNP weight ratio of 1:0.1 was used in the preparation of all formulations. The atomization gas flow rate was set at 600 L/h (55 mmHg), aspirator rate at 35 m^2^/h (100%), inlet temperature at 125 °C, and pump rate at 100%. For drug-loaded CMC nano-/microgel preparation, 10% *w*/*w* Suramin was added directly to the CMC/GNP solution. The spray-drying conditions are summarized in Table 2.

### 4.2. Water Content in Dry Powder

The moisture content of the dry powder was measured using the Karl Fischer titration method on a TitroLine 750 trace titrator (SI Analytics, Weilheim, Germany), with protocols adapted from previously published methods [50]. To summarize, roughly 5 mg of spray-dried powder was carefully weighed and added directly to the titration vessel containing Hydranal^®^ Coulomat AD reagent. The equipment automatically determined the water content and presented the results as weight percent (% *w*/*w*) of water in the powder. All measurements were performed in triplicate (*n* = 3).

### 4.3. Scanning Electron Microscopy (SEM)

The surface morphology and particle size of the spray-dried powders were examined using a scanning electron microscope (Inspect S, FEI, Brno, Czech Republic). Samples were mounted onto aluminum stubs using double-sided adhesive carbon tabs (Ted Pella, Inc., Redding, CA, USA) and sputter-coated with a thin layer of gold using a Hummer 6.2 sputtering system following previously reported research work [51]. Imaging was performed at an accelerating voltage of 15.0 kV. Representative micrographs were captured from multiple regions of each sample at various magnifications to assess particle morphology and surface features.

### 4.4. Laser Diffraction Particle Size and Size Distribution

The particle size and size distribution of the spray-dried carboxymethyl chitosan (CMC) swellable microgels were determined by laser diffraction using a SALD-7101 particle size analyzer (Shimadzu, Kyoto, Japan). For analysis, 10 mg of dry CMC microgel powder were dispersed in 1 mL of Milli-Q water and sonicated for 5 min to ensure homogeneous dispersion. The suspension was transferred into the measurement cell containing water as the dispersant medium, and a refractive index of 1.60–0.10 was applied. The instrument reported the volume-based particle size distribution parameters Dv10, Dv50, and Dv90. The span value, indicating the width of the distribution, was calculated as follows:

$$Span=\frac{Dv90-Dv10}{Dv50}$$

### 4.5. Structural Characterization via X-Ray Diffraction

The crystallinity of the spray-dried powders was determined using X-ray powder diffraction on a D8 Quest diffractometer (Bruker Corporation, Bremen, Germany). Diffraction patterns were obtained at room temperature with an angular range of 5.0° to 50.0° (2θ) and a scan rate of 2.00°/min. To reduce background noise, the samples were placed on a zero-background silicon sample holder. The data were evaluated to determine if the formulations contained crystalline or amorphous domains.

### 4.6. Differential Scanning Calorimetry (DSC)

Differential scanning calorimetry was used to assess the thermal behavior and phase transitions of co-spray-dried powders. Approximately 5–10 mg of each sample was carefully weighed and sealed in anodized aluminum hermetic pans. Thermal analysis was performed using a Q1000 DSC equipment (TA Instruments, New Castle, DE, USA) throughout a temperature range of −5 °C to 300 °C, with a heating rate of 5 °C/min and a constant supply of nitrogen gas. To ensure reproducibility, all measurements were performed three times. All measurements were conducted in triplicate (*n* = 3).

### 4.7. Birefringence and Phase Transitions via Hot-Stage Microscope

Thermal transitions and variations in birefringence were observed using hot-stage microscopy on co-spray-dried CMC-based microgel powders. A small amount of powder was equally placed on a glass microscope slide and heated according to the same temperature protocol as in the DSC study. The observations were conducted using a Leica DMLP microscope (Wetzlar, Germany) with a Mettler FP80 central processor and FP82 hot stage (Columbus, OH, USA) under cross-polarized light. The pictures were taken with a Nikon Coolpix 8800 camera (Nikon, Tokyo, Japan) at 10× optical magnification and 10× digital zoom.

### 4.8. Morphology Observation

The swelling behavior of the spray-dried CMC-based microgels was assessed after incubation under simulated release conditions. A total of 20 mg of dry powder was dispersed in 2.0 mL of PBS, pH 7.4, and incubated at 37 °C for 60 min. After incubation, a drop of the microparticle suspension was placed on a glass slide for observation under an inverted optical microscope (Zeiss Axio Vert.A1, Carl Zeiss Microscopy, Jena, Germany) equipped with a digital camera (AxioCam ERC 5s). The particle diameter was determined by image analysis using Zen 2.3 lite software (Jena, Germany).

### 4.9. In Vitro Drug Diffusion Studies Using the Franz Cell

A modified Franz diffusion cell (VB6; PermeGear Inc., Hellertown, PA, USA) equipped with a Spectra/Por^®^ membrane disc (12–14,000 MW) was used to assess the drug dissolution rate of the Suramin-loaded DP. In short, 5 mL of PBS at 37 ± 0.5 °C was used as the acceptor medium. Using a dry powder Insufflator™Model DP-4M (Penn-Century, Wyndmoor, Philadelphia, PA, USA), 5 mg of dry powder was aerosolized and placed in the donor vessel. A 0.2 mL sample was taken out and replaced with new medium at the appropriate time intervals. HPLC was used to analyze each sample. All experiments were performed in triplicate (*n* = 3).

### 4.10. In Vitro Aerosol Dispersion Performance

The in vitro aerosol dispersion investigation followed US Pharmacopeia (USP) Chapter <601> specifications for aerosols. The Next Generation Impactor^®^ (NGI^®^) (Copley Scientific, Nottingham, UK) with a stainless steel induction port (USP neck) connection was employed at a flow rate of 60 L/min and an actuation time of 10 s through the inhaler device. The aerosol dispersion performance was evaluated with an FDA-approved Aerolyzer^®^ (Merck, Whitehouse Station, NJ, USA) DPI. Quali-V clear HPMC size 3 inhalation-grade capsules (Qualicaps, RTP, NC, USA) contained approximately 10 mg of powder. Critical quality attributes of aerosols include fine particle dose (FPD), respirable fraction with respect to emitted dose (RF), and emitted dose (ED). The fine particle fraction was defined as the proportion of the mass of particles deposited in stages 2–7 to the total recovered mass in all NGI stages, including induction port (FPF). One-way analysis of variance (ANOVA) was performed to Aerodynamic performance of SD CMC microparticles. When statistically significant differences were detected (*p* < 0.05), Tukey’s post hoc test was applied to identify pairwise differences between formulations. Statistical computations were carried out using SPSS version 27 (IBM).

### 4.11. In Vitro Human Pulmonary Cell Viability

The cytocompatibility of CMC-based swellable microgels was evaluated using the resazurin reduction assay. H441, human papillary lung adenocarcinoma cells (ATCC^®^ HTB-174™) and H358, human bronchioalveolar epithelial cells (ATCC^®^ CRL-5807™) were purchased from the American Type Culture Collection ATCC^®^ (Manassas, VA, USA) and were selected due to their relevance for in vitro pulmonary toxicity testing. Cells were maintained in Dulbecco’s Modified Eagle’s Medium (DMEM) supplemented with Advanced 1×, 10% (*v*/*v*) fetal bovine serum (FBS), 100 U/mL penicillin, 100 µg/mL streptomycin, 0.5 µg/mL amphotericin B (Fungizone), and 2 mM L-glutamine in a humidified incubator at 37 °C with 5% CO_2_.

Cells were seeded in 96-well plates at a density of 5000 cells/well in 100 µL of complete medium and allowed to attach for 48 h. After attachment, the culture medium was replaced with fresh medium containing different concentrations of CMC microgel formulations (1–1000 µM), dispersed in DMEM. After 48 h of incubation, 20 µL of a 20 µM resazurin sodium salt solution was added to each well and incubated for an additional four h. The fluorescence was measured using a Synergy H1 Multi-Mode Reader (BioTek Instruments, Winooski, VT, USA) at 544 nm excitation and 590 nm emission. Experiments were performed in triplicate (*n* = 3).

## Figures and Tables

**Table 1:** Aerodynamic performance of SD CMC microparticles with increasing L-leucine content, with or without suramin. Data are expressed as mean ± SD. Reprinted from Encinas-Basurto, McCombs, Vallorz and Mansour [13]. Different letters within the same column indicate statistically significant differences according to Tukey’s test (*p* < 0.05).

System Composition (% Leu *w*/*w*)	FPF < 5 µm (%)	RF (%)	MMAD (µm)	GSD
0	13.5 ± 1.8 a	37.7 ± 4.9 a	11.3 ± 1.8 d	3.0 ± 0.1
2.5	20.4 ± 2.3 b	55.8 ± 0.9 b	5.9 ± 0.4 c	3.1 ± 0.1
5	29.4 ± 0.9 c	75.2 ± 2.0 c	4.1 ± 1.1 bc	3.1 ± 0.7
10	35.2 ± 1.0 d	85.3 ± 1.3 d	1.0 ± 0.4 a	3.4 ± 0.3
10 with Sur	34.4 ± 2.79 d	81.9 ± 0.63 d	2.07 ± 0.3 ab	2.85 ± 0.3

**Table 2:** Spray-drying conditions and residual moisture content of CMC-based microparticles with increasing L-leucine concentrations, with or without suramin.

Powder Composition	Feed Concentration (% *w*/*v*)	Pump Rate (%)	Inlet T (°C)	Outlet T (°C)	Karl Fisher (%)
Spray Dry CMC *w*/*v* 0% Leu	0.2	25	125	49	16.75 ± 0.50
Spray Dry CMC *w*/*v* 2.5% Leu	0.2	25	125	49	18.73 ± 0.65
Spray Dry CMC *w*/*v* 5% Leu	0.2	25	125	49	19.76 ± 0.96
Spray Dry CMC *w*/*v* 10% Leu	0.2	25	125	49	23.63 ± 1.37
Spray Dry CMC/Suramin *w*/*v* 10% Leu	0.2	25	125	49	17.77 ± 0.85

## Data Availability

The original contributions presented in the study are included in the article; further inquiries can be directed to the corresponding authors.

## Abbreviations

The following abbreviations are used in this manuscript:

CMC	Carboxymethyl Chitosan
GNP	Genipin
SEM	Scanning Electron Microscopy
DSC	Differential Scanning Calorimetry
XRD	X-ray Diffraction
HSM	Hot-Stage Microscopy
NGI	Next Generation Impactor
MMAD	Mass Median Aerodynamic Diameter
FPF	Fine Particle Fraction
RF	Respiratory Fraction
DPI	Dry Powder Inhaler
Dv10, Dv50, Dv90	Volume-based diameter at 10%, 50%, and 90% cumulative volume
